# Diverse Neuron Properties and Complex Network Dynamics in the Cerebellar Cortical Inhibitory Circuit

**DOI:** 10.3389/fnmol.2019.00267

**Published:** 2019-11-07

**Authors:** Francesca Prestori, Lisa Mapelli, Egidio D’Angelo

**Affiliations:** ^1^Department of Brain and Behavioral Sciences, University of Pavia, Pavia, Italy; ^2^IRCCS Mondino Foundation, Pavia, Italy

**Keywords:** cerebellar cortex, inhibitory interneurons, dynamic properties, cellular neurophysiology, synaptic inhibition

## Abstract

Neuronal inhibition can be defined as a spatiotemporal restriction or suppression of local microcircuit activity. The importance of inhibition relies in its fundamental role in shaping signal processing in single neurons and neuronal circuits. In this context, the activity of inhibitory interneurons proved the key to endow networks with complex computational and dynamic properties. In the last 50 years, the prevailing view on the functional role of cerebellar cortical inhibitory circuits was that excitatory and inhibitory inputs sum spatially and temporally in order to determine the motor output through Purkinje cells (PCs). Consequently, cerebellar inhibition has traditionally been conceived in terms of restricting or blocking excitation. This assumption has been challenged, in particular in the cerebellar cortex where all neurons except granule cells (and unipolar brush cells in specific lobules) are inhibitory and fire spontaneously at high rates. Recently, a combination of electrophysiological recordings *in vitro* and *in vivo*, imaging, optogenetics and computational modeling, has revealed that inhibitory interneurons play a much more complex role in regulating cerebellar microcircuit functions: inhibition *shapes* neuronal response dynamics in the whole circuit and eventually regulate the PC output. This review elaborates current knowledge on cerebellar inhibitory interneurons [Golgi cells, Lugaro cells (LCs), basket cells (BCs) and stellate cells (SCs)], starting from their ontogenesis and moving up to their morphological, physiological and plastic properties, and integrates this knowledge with that on the more renown granule cells and PCs. We will focus on the circuit loops in which these interneurons are involved and on the way they generate feed-forward, feedback and lateral inhibition along with complex spatio-temporal response dynamics. In this perspective, inhibitory interneurons emerge as the real controllers of cerebellar functioning.

## Cerebellar Interneurons Classification

### Circuit Microanatomy

**Figure 1:**
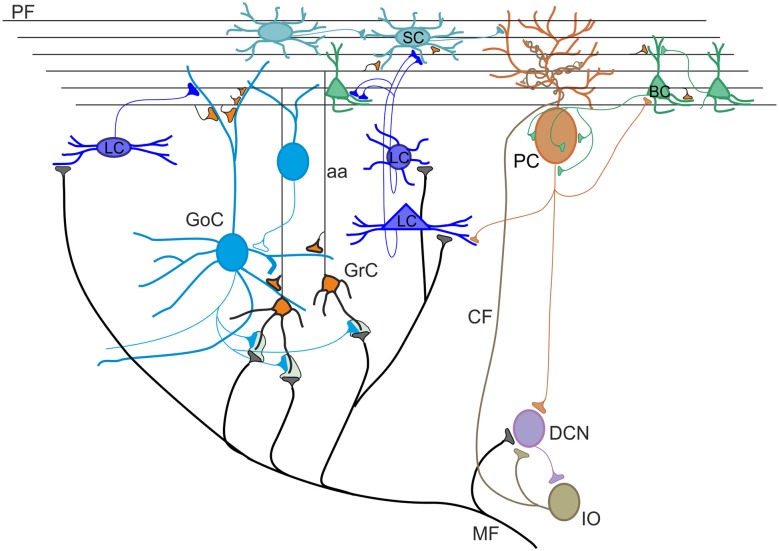
Schematic view of the cerebellar circuit. All cells in the cerebellar cortex are inhibitory except granule cells and unipolar brush cells (not shown). The cerebellar cortex receives two excitatory inputs from mossy fibers (MF) originating in various brain stem and spinal cord nuclei and from climbing fibers (CF) originating from the inferior olive (IO). Mossy fibers contact the granular layer [GL; containing granule cells (GrC), Golgi cells (GoC) and Lugaro cells (LC)] and the deep cerebellar nuclei (DCN). Climbing fibers contact Purkinje cells (PC) and DCN. The ascending axon (aa) of the GrC bifurcates in the molecular layer (ML) forming the parallel fibers (PF), which synapse onto PCs and ML interneurons [stellate cells (SCs) and BCs]. The only output of the cortex is provided by PCs, which project to the DCN. The activity of PCs is under inhibitory control by SC and BC. SC and BC mutually inhibit each other and are coupled through gap junctions. Modified from D’Angelo et al. (2016).

The cerebellar cortex consists of three layers, namely the molecular layer (ML), the Purkinje cell layer (PCL) and the granular layer (GL; Figure 1). The primary input systems enter the cerebellum *via* mossy fibers (MFs) and climbing fibers (CFs). Both are excitatory and use glutamate as neurotransmitter. The MF input originates from several nuclei in the brain stem and spinal cord. In the GL, MFs make excitatory synapses onto granule cells, whose axons rise vertically to the ML, where they divide to form T-shaped branches called parallel fibers (PFs; Pijpers et al., 2006; Oberdick and Sillitoe, 2011). Each PF makes excitatory contacts with hundreds of Purkinje cells (PCs) that, in turn, make inhibitory synapses onto deep cerebellar nuclei (DCN) neurons. At the same time, DCN sends excitatory and inhibitory fibers to the cerebellar cortex generating a positive internal feedback (Ankri et al., 2015; Gao et al., 2016). The PCs provide the only output of the cerebellar cortex. Since PCs are GABAergic, the control exerted on DCN neurons is inhibitory. In addition, DCN neurons receive excitatory synaptic contacts from mossy and CFs collaterals. The CF input originates from the inferior olive (IO). Each PC receives a strong excitatory input *via* a single CF (Ito, 2013; Ito et al., 2014). The inhibitory control exerted by PCs on DCN neurons can be powerfully modulated by local inhibitory circuits formed by basket and stellate cells (SCs). These latter receive excitatory synapses from PFs and inhibitory synapses from PC axon collaterals (Crook et al., 2007; Witter et al., 2016). Basket cells (BCs) are found in the deep ML and provide a powerful inhibitory input to PC bodies and axonal initial segments. SCs are located in the upper ML and make synaptic contacts on PC dendrites, determining a weaker inhibitory influence since they contact the PCs more distally compared to BCs. In the GL, there are two types of interneurons, characterized by a mixed glycinergic/GABAergic phenotype, which do not directly regulate the efferent activity of PCs: Lugaro cells (LCs) and Golgi cells. The LCs are located just beneath the PCL and are the primary target of serotonin released from extracerebellar fibers (Lainé and Axelrad, 1998). Their axons contact basket and SC soma and dendrites in the ML and, through collaterals, form a major input to Golgi cells (Dieudonné and Dumoulin, 2000). In addition, LC soma and dendrites appear to be densely innervated by PC axon collaterals (Lainé and Axelrad, 2002; Crook et al., 2007; Witter et al., 2016). Golgi cell bodies lay in the GL. They receive a double excitatory input: on the basal dendrites from MFs and ascending granule cell axons, and on the apical dendrites from PFs (Chan-Palay et al., 1977; Dieudonné, 1998; Vos et al., 1999). Recently, several lines of evidence for functional gap junctions and chemical synapses among Golgi cells were provided (Dugué et al., 2009; Vervaeke et al., 2010; Hull and Regehr, 2012; Eyre and Nusser, 2016; Szoboszlay et al., 2016). Golgi cell axon occupies the GL and inhibits, in turn, granule cell dendrites (Hámori and Szentágothai, 1966). Lastly, the candelabrum cells, first described in 1994 by Lainé and Axelrad (1994) in the rat, are located within the PCL. They have one or two thick dendrites, dividing into few branches, which run almost vertically into the ML, and several short dendrites which spread for a short distance into the granule cell layer. The connectivity and the function of candelabrum cells have not been investigated yet, though their dendritic structure suggests that PFs and CFs might provide afferent inputs. Current evidences indicate that these cells use GABA and glycine as transmitters (Flace et al., 2004; Tanaka and Ezure, 2004; Crook et al., 2006).

### Embryological Origin and Development

**Figure 2:**
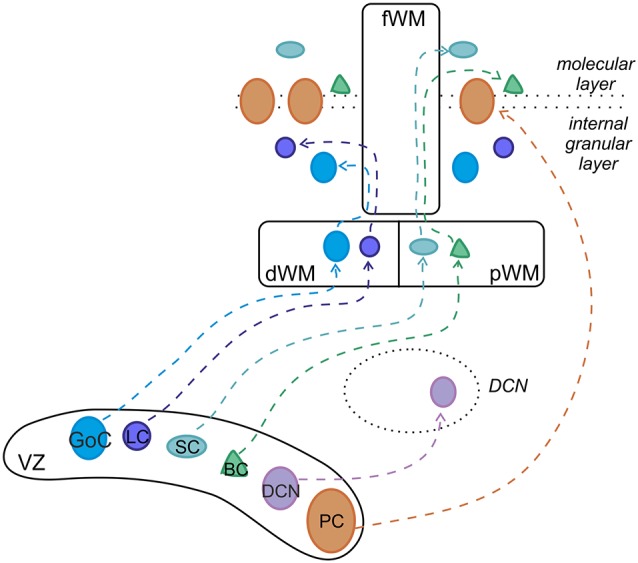
Developmental origin of cerebellar GABAergic neurons. *Ptf1-a*-positive progenitors in the ventricular zone (VZ) generate the entire repertory of GABAergic projection neurons and interneurons through different neurogenic strategies. While projection neurons proliferate and become specified within the VZ, cortical interneurons derive from precursors that originate in the VZ but continue their neurogenic activity in the pWM or dWM, where they acquire mature identities under the influence of specific extracellular cues. Afterward, the interneurons move to their final destination through the folial white matter (fWM). pWM, prospective white matter; dWM, deep white matter; fWM, folial white matter; DCN, deep cerebellar nuclei; PC, Purkinje cell; BC, basket cell; SC, stellate cell; LC, Lugaro cell; GoC, Golgi cell. Modified from Leto et al. (2012).

All cerebellar neurons arise from two primary germinal epithelia: the ventricular zone (VZ) gives origin to GABAergic neurons, whereas the rhombic lip (RL) generates glutamatergic types (Altman and Bayer, 1997; Figure 2). GABAergic DCN neurons are produced first, followed by PCs. Within the VZ, these projection neurons proliferate and acquire specific mature phenotypes through cell-autonomous programs (Florio et al., 2012). By contrast, cortical interneurons, including basket, stellate, Golgi and LCs, derive from precursors that continue their neurogenic activity in a secondary germinative zone i.e., the prospective white matter (pWM) or the deep white matter (dWM), where they differentiate in mature identities under the influence of specific extracellular signals (Leto et al., 2006, 2009, 2012; Leto and Rossi, 2012). In regard to gene expression, the basic helix-loop-helix (bHLH) transcription factor *Ptf1a* is crucial for the initial specification of the GABAergic lineage, ensuring the appropriate cell number production and their subsequent survival (Hoshino et al., 2005; Hoshino, 2006; Pascual et al., 2007; Dennis et al., 2019). Recent studies have shown that, in *Ptf1a* KO mice, GABAergic interneurons adopt a glutamatergic fate, characteristic of external granular layer (EGL) cell precursors (Glasgow et al., 2005; Pascual et al., 2007; Hori et al., 2008), indicating that *Ptf1a* is also necessary for the suppression of the granule cell phenotype in VZ-derived progenitors. While it is clear that *Ptf1a* expression directly specifies GABAergic or glutamatergic neural fate, it has not yet been understood whether all GABAergic types are actually generated from a single pool of VZ progenitors. Several reports have established that the VZ includes different microdomains, characterized by specific gene expression profiles, which are believed to be the origin of different populations of GABAergic neurons (Chizhikov et al., 2006; Sillitoe and Joyner, 2007; Zordan et al., 2008; Lundell et al., 2009; Sudarov et al., 2011). Among the VZ microdomains, some are known to give rise to different classes of PCs, while others are associated with the generation of interneurons (Zordan et al., 2008; Lundell et al., 2009). To date, the only cerebellar GABAergic interneuron-specific marker is *Pax-2* (Maricich and Herrup, 1999; Weisheit et al., 2006). The first *Pax-2*-positive cells appear at E12.5, continue to proliferate up to the birth and then originate major types of GABAergic interneurons through a precise inside-out sequence (Zhang and Goldman, 1996a,b; Altman and Bayer, 1997; Schilling, 2000; Leto et al., 2006): first in the GL (Golgi and LCs), then in the ML (basket and SCs). In the mouse, the generation of 75% of all the interneurons occurs prior to P7 while, in the rat cerebellum, it is completed within the second postnatal week (Weisheit et al., 2006; Leto et al., 2008). The mechanism by which *Pax-2* regulates the GABAergic fate of cerebellar neurons is not completely clarified. *Ptf1a*-positive progenitors promote distinct genetic cascades to express other proneural genes (Zordan et al., 2008; Consalez and Hawkes, 2012; Dastjerdi et al., 2012) which are suitable candidates to operate as determinants of GABAergic identity. Indeed, VZ cells are characterized by the expression of *neurogenin-1* (*Ngn-1*), *neurogenin-2* (*Ngn-2*), and *Ascl-1* genes (Zordan et al., 2008). Precursors expressing *Ngn-1* give rise to PCs (Kim et al., 2008; Lundell et al., 2009; Leto and Rossi, 2012) while only a defined subset of PCs and GABAergic DCN neurons derive from *Ngn-2*-positive progenitors (Florio et al., 2012). Finally, all GABAergic interneurons of cerebellar cortex derive from *Ascl-1* precursors (Kim et al., 2008; Grimaldi et al., 2009; Sudarov et al., 2011). During development, a four-layered organization is evident in the cerebellar cortex: the EGL, the ML, the PCL and the internal granular layer (IGL; Sillitoe and Joyner, 2007). By the third postnatal week, the EGL completely disappears and the IGL becomes the GL (Zhang and Goldman, 1996a; Maricich and Herrup, 1999). From VZ, Golgi cells progenitors migrate to reach the dWM while continuing to undergo cell division. Afterward, through the folial white matter (fWM), they move to their final destination within the IGL until around P4 (Zhang and Goldman, 1996a; Maricich and Herrup, 1999; Weisheit et al., 2006; Galas et al., 2017). Additionally, a specific population of Golgi cells, indentified as *Zac1*-positive, are derived from EGL overlying posterior lobules IX and X. Compared to Golgi cells migrating from VZ, they show a different birthdate time window. LCs derive from progenitors in the dWM, and move through the fWM to reach their final location at the top of the IGL (Galas et al., 2017). At this time, there are contradictory results concerning birthdate time and differentiation of LCs. In rats, mature LCs were suggested to appear towards the end of the second postnatal week (Altman, 1972; Altman and Bayer, 1997) but Lainé et al. (1992) have shown that LCs differentiate at a much earlier age (around P5) suggesting a postnatal migration to the IGL. Basket and SCs migrate from VZ to pWM and postnatally through the fWM while continuing to divide until the second postnatal week (Zhang and Goldman, 1996a; Carletti and Rossi, 2008; Cameron et al., 2009; Galas et al., 2017; Wefers et al., 2018). Subsequently, they move radially to accumulate at the inner border of EGL and then tangentially to reach their final location within the ML.

### Localization and Distribution of Neurochemical Markers

Since the 1980s, with the advent of immunohistochemical strategies for identifying cell types, researcher had begun correlating cell-specific neurochemical marker expression (typically Ca^2+^-binding proteins, neuropeptides and certain receptors) with morphological and electrophysiological characterization in order to distinguish several subtypes of GABAergic interneurons (Kubota et al., 1993, 2011; Kubota and Kawaguchi, 1994, 1997; Cauli et al., 2014). Recent advances suggest that neurons expressing an unidentified cytoplasmic antigen Rat-303 (Hockfield, 1987; Hockberger et al., 1994; Geurts et al., 2001), the metabotropic glutamate and serotonin receptors (Neki et al., 1996; Geurts et al., 2001, 2002; Simat et al., 2007; Sillitoe et al., 2008), the neuropeptide somatostatin (SOM; Johansson et al., 1984; Geurts et al., 2001; Galas et al., 2017) and Ca^2+^-binding proteins such as parvalbumin and calretinin, respectively (PRV and CRT; Schneeberger et al., 1985; Rogers, 1989; Geurts et al., 2001; Schwaller et al., 2002; Bastianelli, 2003; Pibiri et al., 2017), account for nearly 100% of cerebellar GABAergic interneurons. Rat-303 antibody selectively stains Golgi cells and LCs (Hockfield, 1987; Rogers, 1989; Dieudonné and Dumoulin, 2000; Geurts et al., 2001, 2002). Large Rat-303-positive cells displaying also mGluR2, 5-HT2A/5HT5A, SOM and neurogranin immunostaining were identified as Golgi cells based on their location and morphology (typically spherical soma with fan-shaped dendritic arborization located in the depth of the GL). Conversely, large cells, located just underneath the PCL and characterized by a fusiform soma, displayed both Rat-303 and CRT-immunoreactivity. These latter were identified as LCs. However, Rat-303 staining in LC was less pronounced that in Golgi cells (Geurts et al., 2001). Moreover, LCs have been shown to be immunopositive for mGluR1α and mGluR5 in rat (Baude et al., 1993; Hámori et al., 1996; Négyessy et al., 1997; Víg et al., 2003), respectively. Besides LCs, mGluR1α immunoreactivity has been described also for Golgi cells and ML interneurons (Baude et al., 1993; Gorcs et al., 1993; Hámori et al., 1996) while CRT antibody, especially in rat and macaque, could stain Golgi cells, although in much lower numbers (Diño et al., 1999; Geurts et al., 2001). Finally, PRV was found in two subpopulations of GABAergic interneurons within the ML, SCs and BCs (Celio, 1990; Kosaka et al., 1993; Geurts et al., 2002). PRV immunolabeling of BCs revealed the staining of “pinceau” formation, i.e., ramified axons of BCs embracing the axon initial segment (AIS) of PCs. Double immunohistochemistry for PRV and 5HT5A showed 5HT5A immunoreactivity in PRV-positive SCs and BCs (Geurts et al., 2002). Developing of new Cre-driver mouse lines, together with viral vector tools, could provide a very useful support to unravel the complexity of GABAergic interneurons, concurrently contributing to promoting considerable advances in the entire field (Taniguchi et al., 2011; Madisen et al., 2012).

Box 1 **How the Concept of Cerebellar Inhibition Evolved.**

The cerebellum has always been considered as a distinct subdivision of the brain. Aristotle in the 4th century BC wrote: “Behind, right at the back, comes what is termed the cerebellum, differing in form from the brain as we may both feel and see” (Thompson, 1908). Over the years, there was an increasingly accurate description of its structural entity and major subdivisions. By the beginning of the 19th century, the classic anatomical studies were completed and experimental investigation of the cerebellar function began. Three researchers, Rolando (1773–1831), Flourens (1794–1867) and Luciani (1840–1919) helped shaping our understanding of the cerebellum through animal studies. Rolando first demonstrated that, following cerebellar injuries, disturbances of voluntary movements occurred (Rolando, 1809). Flourens observed that cerebellar ablation altered the “harmony of coordinated movements” (Flourens, 1824). Luciani described the three classical symptoms (atonia, asthenia and astasia) of cerebellar diseases (Luciani, 1907). The first description of the functional organization of the cerebellar cortex was proposed in 1906 by the Dutch anatomist Lodewijk Bolk, who divided the cerebellum into four main regions: the anterior lobe, the posterior vermis, and the paired cerebellar hemispheres. He was the first to conceive a functional localization for the coordinating action of the cerebellum in the motor system (Bolk, 1906; Voogd and Koehler, 2018). It was in the later parts of the 20th century that neuronal mechanisms of cerebellar functions were extensively investigated, employing the vestibulo-ocular reflex (VOR) adaptation, eye-blink conditioning and learning in arm movements as experimental paradigms (Ito, 2002), in association with a careful electrophysiological characterization of neurons. The main concepts emerged that *the cerebellar cortex was dominated by inhibitory neurons, whose function was regulated by long-term synaptic plasticity*. The enormous successes reported by these researchers has determined a significant shift in our knowledge of cerebellar cortex circuitry, leading to characterize all inhibitory interneurons and their functional connections and plasticities (for comprehensive reviews, see Hansel et al., 2001; D’Angelo, 2014; Mapelli et al., 2015; D’Angelo et al., 2016; Gao et al., 2016).

**Figure 3:**
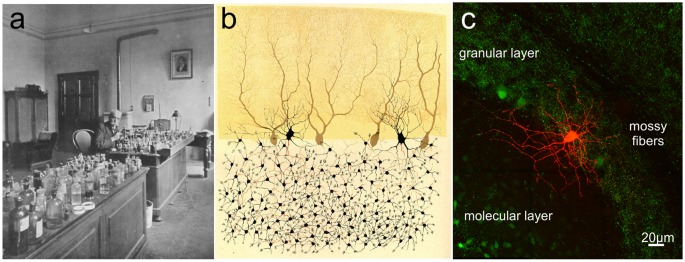
Camillo Golgi and the cerebellar cortex. **(A)** Camillo Golgi in his laboratory at the University of Pavia. **(B)** Illustration by Camillo Golgi of a Golgi impregnated preparation of the cerebellum. Taken from Golgi (1883; available *via* license CC BY 4.0). **(C)** The current high-resolution rendering of a Golgi cell filled with a fluorescent dye and imaged with a two-photon microscope (courtesy of J. DeFelipe).

It is impossible to discuss the history of cerebellar interneurons research without referring to Camillo Golgi (Figure 3) and Santiago Ramon y Cajal. In 1873, Golgi (1843–1926) described two distinct categories of neurons in the granular layer (GL), which were named Golgi Type I and Golgi Type II (Golgi, 1873). It is probable that the first type was the Lugaro Cell [LC; this name derives from the first detailed description of these cells by Lugaro (1894)], whereas, type II corresponded to a neuronal population that later was called by Cajal (1894) the Golgi cells. Still today, the main feature to identify the Golgi cell is the broad extension of its considerable axonal plexus (Dieudonné, 1998; Forti et al., 2006), so well illustrated by Golgi himself. Moreover, he tried to identify a role for these cells: since Golgi cell axonal plexus do not extend beyond the cerebellar cortex, Golgi speculated that they were connectional elements in the network (Golgi, 1873). During the first 60 years of the 20th century, no additional findings were added to clarify Golgi cell physiological function. An important advancement occurred in the 1964 when Eccles (1903–1997) discovered that Golgi cells operate a double feedforward and feedback inhibition of the granule cells (Eccles et al., 1964; Eccles, 1967), contributing to provide the first evidence of a central inhibitory neuron. This result led to the definition of two theories: John Eccles elaborated the Beam Theory (Eccles, 1967, 1973) and Marr (1945–1980) the Motor Learning Theory (Marr, 1969; Albus, 1971). Eccles proposed that Golgi cells, causing a strong inhibition in granule cells, would improve the spatial discrimination of the inputs reaching the cerebellar cortex. Instead, Marr predicted that Golgi cells would be capable to regulate GL excitability and, thus, the amount of information that can be elaborated, transmitted and learned (Marr, 1969). Although both theories were quite appealing and appeared to provide an exhaustive explanation for the whole cerebellum and Golgi cells functions, electrophysiological recordings *in vitro* and *in vivo*, in the 1990s redefined the connectivity of these neurons and their histochemical and functional properties (see below and for review D’Angelo, 2016, 2018). Cajal (1854–1934), by applying Golgi staining to the cerebellum, confirmed the cell types that Golgi had identified and added a detailed morphological characterization of all the elements of the cerebellar cortex, including stellate cells (SCs) and basket cells (BCs) as we know them today (Cajal, 1888). His great contribution was not properly exploited until the 1960s when Rodolfo Llinas characterized and defined the excitatory and inhibitory nature of all synaptic interactions within the cerebellar cortex (Eccles et al., 1966a). He demonstrated that all connectivities in the cerebellar cortex were inhibitory with the exception of the mossy fiber (MF)-granule cell-parallel fiber (PF) system and climbing fiber (CF) input (Eccles et al., 1966b) Moreover, Llinas’s experiments showed that the stimulation of PFs excited molecular layer (ML) interneurons and evoked in Purkinje cells (PCs) an early excitatory postsynaptic potential (EPSP) followed by disynaptic and prolonged inhibitory postsynaptic potentials (IPSPs) that were strongly dendritic as well as somatic (Eccles, 1967). These results were against the prevailing dogma that the soma is considered to be the only location for inhibition. Thus, dendritic inhibition started to represent a different view of neuronal integration. It can therefore be safely concluded that *the study of cerebellar inhibitory mechanisms as contributed not just to understand the functional mechanisms of the cerebellum but also inhibition in brain circuits as a whole*.

### Morphology and Intrinsic Properties

Given the heterogeneity of GABAergic interneurons, it is crucial to convey the diversity into functional specificity (Gupta et al., 2000; Ascoli et al., 2008; DeFelipe et al., 2013; Kepecs and Fishell, 2014; Zeng and Sanes, 2017). In order to better describe the diverse population of interneurons, several parameters defining axonal and dendritic geometry and intrinsic properties have been used in classification studies. Therefore, the morphological and electrophysiological characterization of GABAergic interneurons reviewed here, together with their synaptic connections, is an important step towards understanding information processing in the cerebellum.

#### Golgi Cells

**Figure 4:**
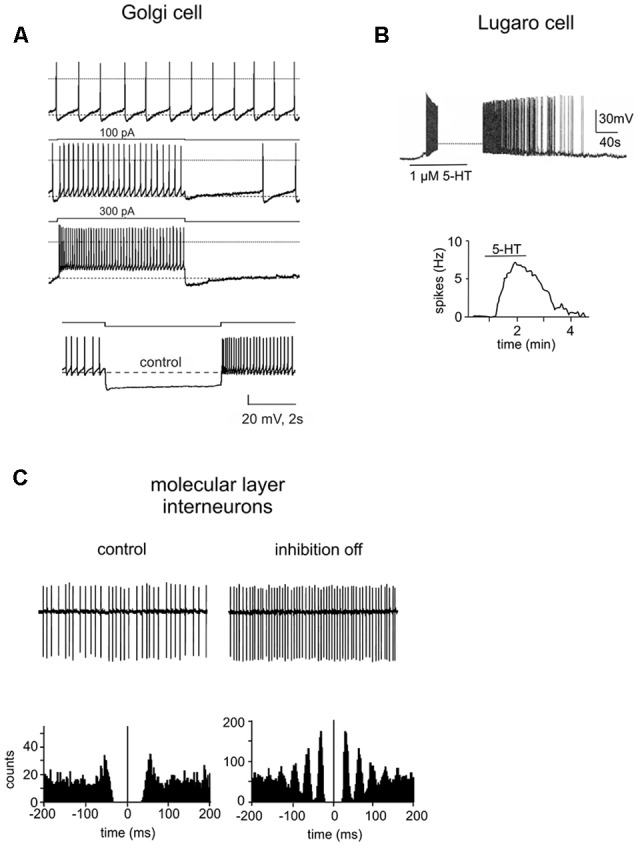
Intrinsic properties of cerebellar GABAergic neurons. **(A)** Electroresponsiveness of a Golgi cell. The neuron shows low-frequency pacemaking activity and, upon depolarizing current injection, high-frequency spike discharge. Spike discharges are followed by an afterhyperpolarization and a silent pause. Upon hyperpolarizing current injection, the Golgi cell shows sagging inward rectification, followed by a post-inhibitory rebound. Adapted from Forti et al. (2006). **(B)** Whole-cell current-clamp recording in rat cerebellar slices demonstrated that the spontaneously inactive LC was reversibly excited by serotonin (1 μM). Below, summary of the effect of serotonin on the firing frequency of a LC. Adapted from Dieudonné and Dumoulin (2000). **(C)** Cell-attached patch-clamp recording from a ML interneuron under control conditions and during the block of inhibition. Below, autocorrelation of action potential trains from the corresponding recordings. Note that the block of inhibition causes a marked increase in the regularity of firing. Adapted from Hausser and Clark (1997).

Golgi cells were first characterized through the pioneering histological studies of Camillo Golgi (Golgi, 1874; Galliano et al., 2010; see Box 1). Golgi cells are the large and primary interneurons located throughout the GL. The majority of them use both GABA and glycine as neurotransmitters (80%) but some use specifically GABA (20%) or glycine (5%; Ottersen et al., 1988; Voogd and Glickstein, 1998; Simat et al., 2007). Immunostainings for different neurochemical markers have underlined the heterogeneity of Golgi cells. Surprisingly, neurogranin labeled GABAergic Golgi cells selectively, whereas mGluR2 was expressed in all Golgi cells with a double neurotransmitter profile (Simat et al., 2007). Golgi cells are characterized by round or polygonal soma emitting from 4 to 10 dendrites (Palay and Chan-Palay, 1974). Golgi cell dendrites can be divided into two classes: basal and apical dendrites. Typically, basal dendrites remain into the GL where they ramify several times acquiring a characteristic curvy appearance. They receive excitatory inputs from MFs and ascending granule cell axons (Cesana et al., 2013). The initial statement that CF collaterals make synaptic contacts on Golgi cell basal dendrites (Hámori and Szentágothai, 1966, 1980; Sugihara et al., 1999; Shinoda et al., 2000) has not been confirmed (Galliano et al., 2013). Although some electrophysiological studies showed that stimulation of CFs caused depression of Golgi cell firing, the nature of this effect still remains to be determined (Schulman and Bloom, 1981; Xu and Edgley, 2008). Apical dendrites, in general stout and straight, ascend towards the ML where they branch extensively forming a distinctive fan-shaped dendritic tree. They receive excitatory synapses *via* PFs of distant granule cells (Chan-Palay et al., 1977; Dieudonné, 1998; Vos et al., 1999; Cesana et al., 2013) and mixed GABA/glycine inputs from LCs (Dumoulin et al., 2001). Pure GABAergic synapses between stellate/BCs and apical Golgi cell dendrites have been also suggested (Palay and Chan-Palay, 1974) but this finding has not been confirmed. Recently, optogenetic studies have shown that axons of stellate/BCs do not functionally innervate Golgi cells and that Golgi cells inhibit each other through reciprocal GABAergic synapses (Hull and Regehr, 2012; Eyre and Nusser, 2016). Finally, apical Golgi cell dendrites are known to form a highly interconnected network using gap junctions endowing Golgi cells with a further level of complexity (Dugué et al., 2009; Vervaeke et al., 2010). Golgi cell axons, composed by very thin beaded fibers, extend profusely into the GL originating widespread neuritic plexi (Dieudonné, 1998; Geurts et al., 2001; Sillitoe et al., 2008). They contribute to the glomerular synapses on granule cell dendrites (Eccles et al., 1966a; Hámori and Szentágothai, 1966; Fox et al., 1967). In the vestibulo-cerebellum, Golgi cell axons make synaptic contact with the unipolar brush cells, in addition to granule cells. Whereas inhibitory postsynaptic responses in granule cells are purely mediated by GABA_A_ receptors, those in unipolar brush cells display a mixed GABAergic/glycinergic component (Dugué et al., 2005; Rousseau et al., 2012). The physiology of Golgi cells has been extensively explored by electrophysiological recordings *in vitro* and *in vivo*. Patch-clamp recordings *in vitro* have reported that (Dieudonné, 1998; Forti et al., 2006; Solinas et al., 2007a,b; Figure 4A):

- Golgi cells are autorhythmic, generating spikes in the range of 1–10 Hz in the absence of synaptic input; occasionally they can be silent during cell-attached recordings.
- they show discharge adaptation during depolarizing current pulses;
- the application of hyperpolarizing current steps determines sagging inward rectification followed by a large rebound depolarization after pulse offset;
- following a sequence of spikes, Golgi cells can reset the phase of their own spontaneous rhythmic firing. After a silent pause lasting exactly as long as the oscillatory period, they restart to discharge;
- Golgi cells are resonant for input frequencies of about 4 Hz.

Rhythmic activity is also observed *in vivo* both in awake and anesthetized animals (Edgley and Lidierth, 1987; Vos et al., 1999; Holtzman et al., 2006a,b; Duguid et al., 2015). It has been recently suggested that gap junction communication between Golgi cells might be essential to allow low-frequency pacemaking and at the same time, to synchronize oscillations in neighboring Golgi cells (Dugué et al., 2009). However, experimental results and simulation with detailed network models have shown that sparse synaptic inputs can tonically and transiently desynchronize Golgi cell networks by triggering a gap junction mediated inhibition (Vervaeke et al., 2010, 2012; Szoboszlay et al., 2016).

#### Lugaro Cells

LCs were first described by Ernesto Lugaro more than a 100 years ago in the cat cerebellum (Lugaro, 1894). They have been characterized as a morphologically distinct GABAergic/glycinergic interneurons with unique physiological features. LCs are mainly distributed in the posterior lobules (VII to X; Lainé and Axelrad, 1996, 1998; Dieudonné and Dumoulin, 2000). On the basis of shape and location of the soma, LCs can be divided into two groups: the first group consists of large-sized LCs characterized by a fusiform or triangular soma which occupy the deeper GL (Lugaro, 1894; Geurts et al., 2001; Melik-Musyan and Fanardzhyan, 2004; Crook et al., 2006). The second group consists of small-sized LCs marked by a fusiform soma located underneath the PCs layer (Lainé and Axelrad, 2002; Simat et al., 2007; Schilling et al., 2008; Hirono et al., 2012). Globular cells, likely a subtype of LCs, have been recently described; they have a small rounded soma and are distributed throughout the GL (Lainé and Axelrad, 2002). From the opposite extremities of the LC soma, two pairs of thick, horizontal, rarely ramified dendrites emerge, running parallel to the PCL in the parasagittal plane (Lainé and Axelrad, 2002; Geurts et al., 2003). These dendrites are very long (from 100 μm to 700 μm) and can ascend obliquely towards the ML and/or extend down more or less vertically through the GL (Lainé and Axelrad, 1996). Conversely, globular cells exhibit radiating dendrites that spread mostly in the PCL (Lainé and Axelrad, 2002; Hirono et al., 2012). LC axons are myelinated and can be divided into two groups, in accordance with their route to the ML (Lainé and Axelrad, 1996). In the first case, the axon is parasagittally oriented and heads downwards in the GL before ascending back and ending inside the ML. This parasagittal plexus makes synaptic junctions with stellate and BC soma. The second axon type enters directly the ML where it generates a local and transversal plexus running parallel to the PFs. These transverse fibers preferentially contact apical dendrites of Golgi cells (Lainé and Axelrad, 1996; Dieudonné and Dumoulin, 2000; Dumoulin et al., 2001). Although LCs have also been suggested to inhibit PCs (Dean et al., 2003), the location of these synapses remains debated (Lainé and Axelrad, 1998, 2002; Simat et al., 2007). Globular cell axons project into the ML following the directly or indirectly ascending trajectory (Lainé and Axelrad, 2002). LCs in the rat cerebellum are normally completely silent (Figure 4B). In the presence of serotonin they become intensively active showing a robust firing (5–15 Hz), determining the inhibition of BCs and SCs, as well as Golgi cells and PCs (Dieudonné and Dumoulin, 2000; Dumoulin et al., 2001; Dean et al., 2003; Hirono et al., 2012). In the cerebellum, the LC-Golgi cell synapse was the first functional evidence of mixed GABA/glycine co-release (Dieudonné, 1995; Dumoulin et al., 2001). Since glycine receptors are not expressed at LC-stellate/basket/PC synapses, LCs can perform target-specific synaptic transmission. High sensitivity to both serotonin and norepinephrine differentiate globular cells from other LCs. Moreover, they receive strong inhibitory synaptic inputs through PC axon collaterals and probably also excitatory synaptic inputs through MFs (Colin et al., 2002; Hirono et al., 2012).

#### Basket Cells and Stellate Cells

BCs and SCs, first described by Golgi (1883) and Cajal (1888) are GABAergic interneurons located in the ML of the adult cerebellum (see Box 1). BCs and SCs have distinctive morphology, positioning and axonal arborization (Lemeky-Johnston and Larramendi, 1968; Chan-Palay and Palay, 1972; Palay and Chan-Palay, 1974; Llinas et al., 2004). BCs have a pyramidal or oval soma with a diameter of 20 μm and are usually found in the inner third of the ML or between PCs. BCs have 4–10 straight dendrites that expand vertically producing a fan-shaped field in the parasagittal plane. In general, the dendrites are large, thick and smooth although some have spines. Many of them, before curving upwards, extend in the horizontal plane above the PCs for millimeters, originating relatively few branches in their course (DeFelipe et al., 1986). SCs, which have a small and fusiform soma about 7–10 μm in diameter, are located in the upper two-third of the ML. They are characterized by long, contorted, aspinous, frequently branching dendrites radiating in all directions. Some cells appear bipolar with dendrites originating from opposite sides of the soma (Palay and Chan-Palay, 1974; Jacobs et al., 2014). The longitudinally arranged PFs cross the dendritic tree of both cell types at right angles, providing the major excitatory synaptic input. In addition, several studies have reported excitatory synapses between basket/SCs and CF collaterals acting exclusively *via* spillover of glutamate from nearby release sites. CFs stimulation resulted in an increased spike firing in both interneurons both *in vivo* (Jörntell and Ekerot, 2002, 2003) and *in vitro* (Hámori and Szentágothai, 1980; Szapiro and Barbour, 2009). Finally, both molecular interneurons receive inhibitory GABAergic contacts, mainly on their soma. BC soma is contacted by PC axon collaterals and axons from other BCs (O’Donoghue et al., 1989; O’Donoghue and Bishop, 1990; Hausser and Clark, 1997) whereas SC soma receives axonal contacts from other SCs (Kondo and Marty, 1998). BC axon emerges either from the soma or from one of the major dendrites extending horizontally in the parasagittal plane above the PC soma for the distance of 500–600 μm (Chan-Palay et al., 1974; Castejon et al., 2001). It emits a succession of collaterals: ascending collaterals headed to the ML; descending collaterals that envelop the soma of several PCs forming the pericellular “basket” that gives the cells their name. Some terminate their course surrounding the initial axon segment of PCs establishing a very complicated axo-axonic synapse called *pinceau*. The axons of SCs are less characteristic: they branch immediately generating short and circumscribed collaterals. This simple arborization contacts the dendrites of PCs (Palay and Chan-Palay, 1974; Ito, 1984). Both ML interneurons fire spontaneously in the range of 1–35 Hz, both *in vitro*, in the absence of external inputs, and *in vivo* (Hausser and Clark, 1997; Carter and Regehr, 2002; Jörntell and Ekerot, 2003; Barmack and Yakhnitsa, 2008). The excitability of BCs and SCs is shown to be modulated by several molecular mechanisms. For example, the firing rate of SCs is dynamically regulated by T-type channel-mediated Ca^2+^ transient through A-type K^+^ channel modulation (Molineux et al., 2005; Anderson et al., 2013; Alexander et al., 2019). Moreover, ML interneurons firing patterns are typically irregular, characterized by a shift toward a more regular rate when inhibitory synaptic currents are blocked (Figure 4C; Hausser and Clark, 1997; Lachamp et al., 2009). This irregularity is presumably due to spontaneous CF activities occurring in irregular patterns. Interestingly, CFs, especially terminating in the same parasagittal bands, tend to display synchrony (De Zeeuw et al., 1997; Lang et al., 1999) determining a more synchronized spiking activity of the interneurons localized in the same microzone. This tendency may be further enhanced by mutual inhibitory contacts (see above) and gap junction communication (Mann-Metzer and Yarom, 1999).

## Cerebellar Interneurons Functional Connectivity

Although the functional connections of principal neurons (PCs and granule cells) have been described in detail for the cerebellar cortex (for critical reviews, see Tank et al., 1988; D’Angelo et al., 1995, 2009, 2016; Silver et al., 1996; Hansel et al., 2001; D’Angelo and De Zeeuw, 2009; D’Angelo, 2014, 2016, 2018; Masoli et al., 2015), attention is only recently starting to shift toward the dynamical interactions among inhibitory interneurons. The correlated morphological and functional analysis of inhibitory interneurons is still representing a major challenge. Indeed, GABAergic interneurons differ in molecular, structural and firing properties, making their functional roles in the microcircuits more complex to understand. The inhibitory interneurons appear to be organized in multineuronal inhibitory chains generating feed-forward, feed-back and lateral inhibition, which are likely to cooperate in determining cerebellar signal processing in a way that is not yet fully elucidated. For the sake of simplicity, in the following section, the functional connectivity of cerebellar cortical interneurons will be elucidated on the basis of these three “classical” types of inhibition mediated by GABAergic interneurons in the cerebellum. We will focus on the recruitment of BCs, SCs, LCs and Golgi cells into these circuit mechanisms.

### Feed-Forward Inhibitory Circuits

In a feed-forward inhibitory circuit, principal cells and inhibitory interneurons simultaneously receive are the same excitatory input. The interneurons inhibits the principal cell with disynaptic delay, thereby narrowing the window for suprathreshold summation of excitatory inputs (Buzsáki, 1984; Pouille and Scanziani, 2001; Blitz and Regehr, 2005; Gabernet et al., 2005; Mittmann et al., 2005; Cruikshank et al., 2007; D’Angelo and De Zeeuw, 2009; Torborg et al., 2010; Najac et al., 2011). Feed-forward inhibition (FFI) is a way of regulating the timing of neuronal responses in many brain regions, enhancing network performance.

#### Parallel Fiber–Molecular Layer Interneurons–Purkinje Cell

In the cerebellum, basket and SCs activated by PFs control the rate and temporal precision of PC spike output using FFI (Eccles, 1967; Hausser and Clark, 1997; Jaeger and Bower, 1999; Mittmann et al., 2005; Barmack and Yakhnitsa, 2008). PCs generate complex spikes in response to CF activity (Davie et al., 2008) and simple spikes which occur spontaneously (Hausser and Clark, 1997; Raman and Bean, 1997) or are driven by PF input (Eccles et al., 1967). Thus, the PC spike output reflects a complex interaction between spontaneous activity, excitatory and inhibitory synaptic inputs from PFs and ML interneurons (Hausser and Clark, 1997; Jörntell and Ekerot, 2002; Santamaria et al., 2007). FFI is mediated by two distinct pathways involving different subcellular segments of PCs. Experimental and computational studies suggest that stellate (dendritic)-type inhibition and basket (somatic)-type inhibition play diverse functional roles and have different postsynaptic effects on PCs (Santamaria et al., 2002, 2007; Santamaria and Bower, 2005; Bower, 2010; Masoli and D’Angelo, 2017). Dendritic FFI contributes to compensate PF excitation in local segments of the PC dendrite resulting in indirect influence on spike output. Specifically, the interaction of PF and SC synaptic inputs on PC spike output is mediated by large intrinsic calcium and calcium-activated dendritic currents which, counterbalancing each other, affect the excitability of the PC dendrite. Consistent with simulation-based studies, experimental results suggest that the temporal balance between dendritic FFI and PF input results in a compensation of calcium currents by calcium-activated potassium currents. This, in turn, does not determine any net current flow and thus no effect of PF activity on the PC spike output (Jaeger and Bower, 1999). In conclusion, dendritic FFI is involved in modulating the “state” of the PC dendrite and this regulation of dynamic balance between voltage-dependent conductances is the explanation of how the PC soma activity typically functions (Jaeger et al., 1997; Jaeger and Bower, 1999; Santamaria et al., 2002; Womack and Khodakhah, 2003; Santamaria and Bower, 2005; Bower, 2010). Conversely, somatic FFI is very effective, rapid and powerful in controlling PC responses. Specifically, this type of inhibition: (i) reduces the time window for summation of independent input pathways; (ii) increases the temporal spike precision of; and (iii) suppresses the response to subsequent inputs (Vincent and Marty, 1996; Mittmann et al., 2005; Barmack and Yakhnitsa, 2008). Recently, the BC *pinceau* has been proposed to cause ephaptic inhibition *via* the current flow surrounding the PC AIS (Blot and Barbour, 2014), confirming the prediction derived by analogy with the Mauthner cell axon cap (Chan-Palay and Palay, 1970; Sotelo and Llinás, 1972). Through the ephaptic *pinceau* effect, BCs, once activated by PFs, determine an extremely fast inhibition of PCs, thus without synaptic delay. This is a very effective mechanism to prevent the simultaneous on-beam exciting action of PFs and to reduce off-beam PC firing through a pure inhibition (Blot and Barbour, 2014). The influence of dendritic and somatic FFI on PCs are predicted to be different along the course of PFs (Santamaria et al., 2007).

Despite the long history of works dedicated to understand cerebellar cellular circuitry and function (Cajal, 1908; Eccles, 1967; Voogd and Glickstein, 1998; Voogd, 2014), the last decade of cerebellar research has discovered additional afferent and efferent contacts that could impact on the ML interneuron processing. The high majority of granule cells ascending axon synapses on PCs (Sultan and Bower, 1998; Apps and Garwicz, 2005) are located in the inner ML (Gundappa-Sulur et al., 1999). A recent hypothesis is that ascending axon synapses can also end on BCs, though this is not confirmed by either physiological or anatomical (Gundappa-Sulur et al., 1999) data. If this was true, then an interesting scenario would appear. The ascending axon of granule cell is invaded very rapidly by the spike (Diwakar et al., 2009; Dover et al., 2016), which then run more slowly along the PFs (0.1 m/s). Thus, excitatory inputs arriving at PCs through ascending axons would be too early to undergo the FFI influence. By contrast, at longer distances, PF inputs can be counterbalanced by dendritic FFI. In conclusion, the fundamental features of the connectivity and therefore the function of the cortical network are rigidly determined by the spatial relationship between neuronal components. Different form of plasticity at PF–ML interneuron and ML interneuron–PC synapses are assumed to increase the variability of PC spike output (Albus, 1971; Dean and Porrill, 2010). Several arguments of evidence converge to sustain the concept that ML interneuron FFI plays a role in controlling the gain and timing of motor learning (Wulff et al., 2009; Heiney et al., 2014; Yamazaki et al., 2015; Jelitai et al., 2016). PC GABA_A_ receptor knock-out mice show significant motor coordination deficits (ataxia). Moreover, the modulation of the firing rate of ML interneurons using optogenetics proved able to drive movement kinematics in awake mice. In eyeblink conditioning, a pause in PC firing occurs after training (Jirenhed et al., 2007). However, PC spontaneous activity has been reported to be independent of PF input (Hausser and Clark, 1997; Cerminara and Rawson, 2004). Consequently, the well-timed reduction of PC spontaneous activity could be explained by an acquired increase in molecular interneuron FFI, which could, therefore, contribute to the mechanisms underlying consolidation of the learned eyeblink response. In conclusion, plastic changes in ML interneuron FFI onto PCs could play a crucial role in controlling the temporal aspects of learned output of the cerebellar cortex (Attwell et al., 2002; Cooke et al., 2004; Jörntell et al., 2010; see also below).

#### Parallel Fiber–Molecular Layer Interneuron–Molecular Layer Interneuron

Since ML interneurons are highly interconnected *via* electrical and chemical synapses, in addition to providing FFI to PCs they also receive FFI from each other (Mittmann et al., 2005; Rieubland et al., 2014). Interestingly, the effect of FFI in interneurons appears to be less powerful than that observed in PCs. This could depend on differences in the intrinsic conductances in interneurons or PCs or, as an alternative, PFs could activate fewer feed-forward inhibitory connections among interneurons than onto PCs. Modeling studies investigating the functional role of ML interneuron mutual inhibition revealed a significant influence on activity of the network by regulating the firing rate and variability of spike timing of ML interneurons and PCs. Lennon et al. (2014) simulated a scenario where synapses between ML interneurons were removed. Following decreased mutual inhibition, ML interneuron firing rates increased. The consequence of the increased ML interneuron firing is an increase of FFI onto PCs, resulting in decreased PC firing rates thus preventing DCN neurons from firing appropriately. Thus, FFI onto ML interneurons could be needed to assure effective motor performance and learning (Walter et al., 2006; Wulff et al., 2009).

#### Parallel Fiber–Molecular Layer Interneuron–Golgi Cell

Anatomical and physiological (Dumoulin et al., 2001) lines of evidence have proposed that, in addition to PCs, ML interneurons recruited by PFs inhibit Golgi cells (Figure 4). However, recent studies reported that Golgi cells are synaptically inhibited by other Golgi cells (Dugué et al., 2009; Galliano et al., 2010; Simões de Souza and De Schutter, 2011) rather than by ML interneurons (Hull and Regehr, 2012), and that they are also connected by gap junctions (Isope and Barbour, 2002; Geurts et al., 2003; D’Angelo and De Zeeuw, 2009; Galliano et al., 2010; Jörntell et al., 2010). At present, the existence of ML interneuron–Golgi cell synapses issue remains controversial.

#### Mossy Fiber–Golgi Cell–Granule Cell

In the GL, Golgi cells generate synaptic inhibition onto granule cells. Granule cells receive excitatory inputs from the MFs which, in turn, excite Golgi cells providing FFI to granule cells (Pouille and Scanziani, 2001; Kanichay and Silver, 2008; D’Angelo and De Zeeuw, 2009; D’Angelo et al., 2013). FFI from Golgi cells acts through two main mechanisms, phasic and tonic. Phasic inhibition consist of synaptic GABA_A_-mediated inhibitory post-synaptic currents and potentials (Wall and Usowicz, 1997; Rossi and Hamann, 1998; Armano et al., 2000), while tonic inhibition is mediated by extrasynaptic GABA_A_ receptors activated by low GABA levels in the extracellular space (Brickley et al., 1996; Wall and Usowicz, 1997; Hamann et al., 2002; Rossi et al., 2003; Farrant and Nusser, 2005; Glykys and Mody, 2007). FFI mediated by phasic inhibition enhances granule cell spike timing precision by narrowing the time window for synaptic integration. In response to a single MF input or brief bursts, phasic inhibition generated by the feed-forward circuit lasts about 4–5 ms and limits the duration of granule cell responses to 1–2 spikes. Interestingly, since MF–granule cell LTP tends to anticipate the emission of the first spike, while LTD does the opposite (Nieus et al., 2006; Mapelli et al., 2014; Nieus et al., 2014), synaptic plasticity contributes with the time window mechanism in regulating information transfer (D’Angelo et al., 2013). Extending a prediction from theoretical network analysis (Medina and Mauk, 2000; De Schutter and Bjaalie, 2001), Golgi cells endow the GL with the properties of a temporal filter determining how bursts are conveyed toward ML and how PFs activate PCs and interneurons (Bower, 2002; Lu et al., 2005). Furthermore, at the PF synapses Golgi cells could also regulate the short- and long-term synaptic plasticity induction by controlling the temporal pattern of spikes generated by granule cells (Isope and Barbour, 2002; Sims and Hartell, 2005). During MF high-frequency activity, like that generated in response to sensory stimulation (Chadderton et al., 2004; Rancz et al., 2007), the time window effect can be momentarily abolished, due to diverse possible mechanisms including: (i) presynaptic decrease of GABA release through GABA_B_ autoreceptors or mGlu receptors expressed on Golgi cell terminals (Mitchell and Silver, 2000a,b; Mapelli et al., 2009); (ii) postsynaptic down-regulation of GABA_A_ currents mediated by GABA_B_ activation (Brandalise et al., 2012); (iii) postsynaptic reduction of an inward rectifier potassium current through GABA_B_ receptors which determines an enhancement of granule cell responsiveness (Rossi et al., 2006); and (iv) reduction of Golgi cell firing through dendritic activation of mGlu2 receptors which enhances an inward rectifier potassium current (Watanabe and Nakanishi, 2003). Additional *in vitro* and *in vivo* studies are needed to clarify the significance of these mechanisms. Otherwise, FFI mediated by tonic inhibition determines a shift of input/output (I/O) relationship by decreasing the membrane resistance of granule cells, leading to a reduction of the excitability (Hamann et al., 2002). This holds confirmed only for constant excitatory inputs. When excitation is mediated by time-varying synaptic inputs, tonic inhibition changes the slope (gain) of the I/O relationship (Chance et al., 2002; Mitchell and Silver, 2003). Thus, FFI mediated by tonic inhibition has a double effect: the shift in I/O relationship configure the level of granule cell excitability, while gain regulation makes the neuron less sensitive to changes in its inputs. In this manner, tonic inhibition would allow providing the appropriate excitability of granule cells and discriminating significant information from background activity eventually reducing signal-to-noise ratio of information transmission in granule cells and of PF input to the molecular interneurons and PCs (Duguid et al., 2012; Mapelli et al., 2014).

### Feed-Back Inhibitory Circuits

In a feed-back inhibitory circuit, the principal cell provides the excitatory input onto the inhibitory interneurons, which, in turn, further inhibit the principal cell. Therefore, feedback inhibition (FBI) plays a general role in locally controlling the excitatory-inhibitory (E/I) balance within a neural circuit (Dieudonné, 1998; D’Angelo and De Zeeuw, 2009; D’Angelo et al., 2013; Feldmeyer et al., 2018).

#### Parallel Fiber–Golgi Cell–Granule Cell

Electrophysiological and morphological experimental results indicate that Golgi cells feedback onto the granule cells (Figure 4). Actually, previous *in vivo* researches reported that an intense stimulation of the PFs caused a decrease of the MF excitatory input transmission to PCs, possibly through the excitation of Golgi cells and successive inhibition of granule cells (Eccles et al., 1964, 1966a; Dieudonné, 1998). Considering PF lengths, a granule cell could excite a Golgi cell at a long distance along the transverse section. Conversely, a Golgi cell will only inhibit the granule cells mostly located in the narrow parasagittal zone occupied by its axon. FBI from Golgi cells onto granule cells mediated by phasic inhibition is critical for generating and sustaining *coherent oscillations* (Maex and De Schutter, 1998; Solinas et al., 2010; Mapelli et al., 2014). Following MF input, Golgi cell and granule cell populations become entrained in a synchronous oscillatory activity, whose basic frequency range from 10 to 40 Hz (Maex and De Schutter, 1998). This could account for the large-amplitude oscillation recorded in the GL of freely moving rats (Pellerin and Lamarre, 1997; Hartmann and Bower, 1998; Courtemanche et al., 2002; Courtemanche and Lamarre, 2005) and monkeys. Furthermore, Golgi cell autorhythmic activity (Forti et al., 2006), SC-Golgi cell synapses (Casado et al., 2000), and Golgi cell-Golgi cell synapses and gap junctions (Vervaeke et al., 2010, 2012; Hull and Regehr, 2012) also participate to originate circuit oscillations (Maex and De Schutter, 1998; D’Angelo and De Zeeuw, 2009; Solinas et al., 2010; D’Angelo et al., 2013). Recently, FBI mediated by phasic inhibition has been shown to be implicated in the phenomenon of resonance in the GL (Gandolfi et al., 2013). The authors showed that the blockade of phasic inhibition prevented oscillations but not resonance (which is just modulated), indicating that the two processes have a complicated and only partially mechanistic relationship with the inhibitory circuit. Finally, a computational model of the GL suggested that tonic inhibition generated by FBI desynchronizes the network, but this effect could be counterbalanced completely by enhancing MF firing rate (Maex and De Schutter, 1998). Thus, tonic inhibition could further have an effect on coherence of distributed signal processing (Singer and Gray, 1995; Semyanov et al., 2004).

#### Lugaro Cell–Molecular Layer Interneurons–Purkinje Cell

**Figure 5:**
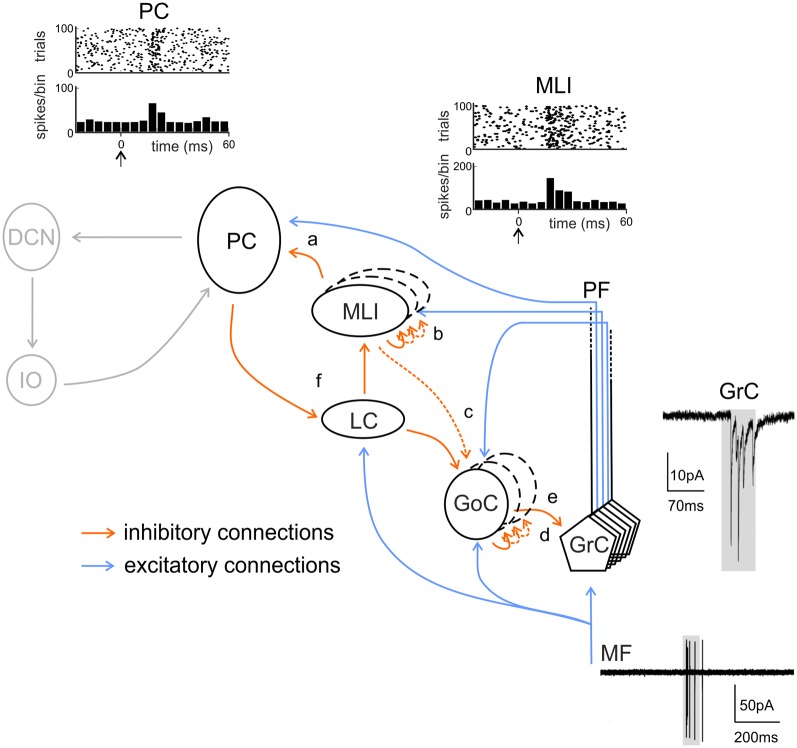
Inhibitory chains in the cerebellar cortex. The scheme shows the main excitatory and inhibitory connections in the cerebellar cortical circuit. Note that feed-forward (FFI) and feed-back (FBI) inhibitory loops are integrated in a complex inhibitory chain. FFI: (a) A PC may be both directly excited and then inhibited with disynaptic delay *via* ML interneurons (MLIs) activated by the same set of active PFs. (b) MLIs receive feed-forward inhibition (FFI) from each other. (c) MLIs, activated by PFs, inhibit Golgi cells (GoCs) in the same manner as PCs. This issue is somewhat controversial (see main text, dashed line). (d) Granule cells (GrCs) receive excitatory inputs from MFs. MFs also excite GoCs which provide FFI to GrCs. FBI: (e) through the PFs, GrCs activate GoCs that, in turn, inhibited GrCs in a feedback loop. (f) PC collaterals are known to inhibit LCs, which in turn inhibit MLIs. Activation of LCs by excitatory MF or monoaminergic inputs would lead to enhanced PC activity through disinhibition. In gray, non-cortical circuits involving DCN and IO. Traces show the activity of MF, granule cells, ML interneurons and PCs during tactile sensory stimulation in rodents. Adapted from Rancz et al. (2007) and Ramakrishnan et al. (2016). Noteworthy, the complex regulatory mechanisms brought about by the inhibitory interneuron chain remain to be investigated during dynamic signal processing.

A LC makes synaptic contacts preferentially with ML interneurons in the sagittal axonal plexus and Golgi cells in the transversal axonal plexus (Simat et al., 2007; Schilling et al., 2008), while soma and dendrites receive massive innervation from PC axon collaterals (Colin et al., 2002; Hirono et al., 2012). LCs, once activated by MFs or monoaminergic inputs, can increase the PC activity through ML disinhibition. Thus, the PC–LC feedback circuit proceeds and could silence LCs. LC activity is able to synchronize the firing of PC clusters in different microzones, likely contributing to motor learning and coordination (Hirono et al., 2012). A previous *in vivo* study described a correlation between the spontaneous firing rate of PCs and the effect of serotonin (Strahlendorf et al., 1984). Specifically, PCs that responded to serotonin with increases in discharge rate showed significantly lower basal firing frequencies than those cells that were silenced by serotonin. This correlation can be explained by FBI circuit. When PCs fire high-frequency action potentials, LCs are allowed to generate only a few spikes even in the presence of serotonin, and PCs firing is no longer facilitated by serotonin, but rather in some cases decreased by the direct effects of serotonin on the PCs (Bishop and Kerr, 1992; Li et al., 1993). Conversely, when PCs fire at low frequencies, serotonin can induce robust firing in LCs, which lead to facilitation of PC firing. At the behavioral level, the pharmacological depletion of brain serotonin in the rabbit causes a loss of vision-guided adaptation of vestibulo-ocular reflex (VOR; Miyashita and Watanabe, 1984). Moreover, a serotonin precursor was used for cerebellar ataxia therapy (Trouillas et al., 1988, 1995). Recent clinical research in patients with ADHD, some of whom show abnormal activity in monoaminergic systems, showed that the timing of conditioned eyeblink responses is impaired (Oades et al., 2008; Frings et al., 2010). In conclusion, since LCs also contact Golgi cells, they may be considered as a key node in modulating inhibition levels both in the molecular and GL (Figure 5).

### Lateral Inhibition

Lateral inhibition is considered a consequence of FBI where a principal neuron response to a stimulus is inhibited by the excitation of a neighboring interneuron by other principal cells nearby. This type of neural network was first discovered by Hartline and Ratliff (1957) in their studies of the compound eye of the horseshoe crab. In general, lateral inhibition enhances neurons responsiveness to spatially varying stimuli than to spatially uniform ones. That is, a neuron stimulated by a spatially uniform stimulus is also inhibited by its surrounding interneurons, thus suppressing its response. By contrast, a neuron subjected to a spatially varying stimulus is less inhibited by its neighbors that are not excited, thus producing stronger response (Bakshi and Ghosh, 2017).

#### Golgi Cell Lateral Inhibition

**Figure 6:**
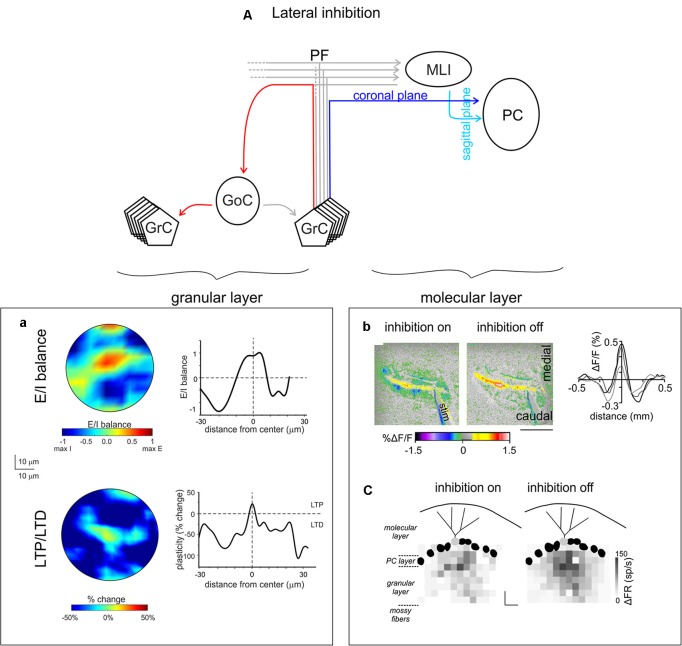
Lateral inhibition in the cerebellar cortex. **(A)** Schematic of the cerebellar circuit of lateral inhibition, with the relevant granular and ML connectivity highlighted at left and at right, respectively. **(a)** GoCs generate a broad lateral inhibition that extends beyond the afferent synaptic field. The center-surround effect is generated by lateral inhibition. After excitation, GrCs in the core are more activated than those in the surrounding area. Below, long-term synaptic plasticity is controlled by Golgi cell synaptic inhibition at the MF-granule cell synapse. The result is LTP in the center and LTD in the surround, so that LTP and LTD assume a center-surround organization. Modified from Soda et al. (2019). **(b)** MLIs provide the substrate for lateral inhibition of PCs by virtue of the orthogonal arrangement of excitation and inhibition onto PCs: the PFs run coronally, whereas the axons of MLIs run sagittally. Surface stimulation of the cerebellar cortex evokes a large on-beam increase in fluorescence attributable to PF excitation of its postsynaptic targets (PCs and MLIs) and a narrow off-beam decrease in fluorescence due to postsynaptic inhibition generated by MLIs. Blocking inhibition using bicuculline application abolishes off-beam decrease in fluorescence and enhances the on-beam increase in fluorescence (scale bar 1 mm). Right, intensity profiles of the fluorescence change perpendicular to the beam. Adapted from Gao et al. (2006). **(c)** Response maps of a single PC (light gray) in terms of change in firing rate (gray scale) with inhibition on or off, while stimulating different regions in the GL. The probability that pure and net inhibitory responses are elicited by granule cells increased as a function of lateral distance from the PC (scale bars 80 μm × 80 μm). Adapted from Dizon and Khodakhah (2011).

Golgi cell primary axonal plexus is confined in the GL and send collaterals originating secondary plexuses in the same or even in nearby laminae (Eccles, 1967; Barmack and Yakhnitsa, 2008). In the case of the Golgi cell, the origin of lateral inhibition phenomena relies on the larger extension of the axonal plexus compared to that of basal dendrites. The functional relevance of this inhibitory organization has recently been described through multi-electrode array recordings and voltage-sensitive dye imaging (Mapelli and D’Angelo, 2007; Mapelli et al., 2009; D’Angelo et al., 2013). Previous investigations *in vitro* have shown that lateral inhibition in the GL originates a *center-surround* organization of granule cell activity (Mapelli and D’Angelo, 2007; D’Angelo, 2008; Soda et al., 2019), characterized by prevailing excitation in the core, surrounded by an inhibited area. The *center-surround* pattern is generated as follows: when the MFs discharge in bursts, both granule cells and Golgi cells are activated in the same region. The resulting E/I balance is characterized by excitation prevailing in the core and inhibition prevailing in the surround, by virtue of the broader inhibitory territory of Golgi cells, with granule cell excitation that decreases radially from the excitation core (Figure 6A; Mapelli and D’Angelo, 2007). A detailed multicompartmental model has calculated that an excited core can contain 260 granule cells with a probability of generating spikes up to 35%; by contrast, this probability is almost zero in the surround. Switching off inhibition in this context increases the probability of firing in the core up to 50% (Diwakar et al., 2009). Therefore, Golgi cells are pivotal players in determining the center-surround organization of granule cell activity following MFs stimulation. Three main functional consequence can be pointed out:

1. Organization of information transmission and processing along channels activating granule cell ascending axons running toward the ML and contacting overlying PCs. Coherently with high excitation levels in the core, the E/I organization in the GL facilitates the transmission of high-frequency burst along the channel. The prevailing inhibition in the surround acts as a filter preventing the transmission of low-frequency discharges (Mapelli et al., 2010). As a consequence, Golgi cells are able to define, converge and refine information transmission to PCs originating transmission channels running vertically to the ML, as suggested by previous experiments (Bower and Woolston, 1983).
2. Dynamic configuration of network topology by controlling the distribution of long-term synaptic plasticity. In particular, the higher excitation level in the core facilitates LTP, while the weaker excitation levels in the surround facilitate LTD. The center-surround organization of the E/I balance determines, in condition of suitable high-frequency stimulation, a matching center-surround distribution of LTP and LTD. This further sharpens the topological organization of signal transmission (Figure 6a). In the perspective of the transmission channeling mentioned above, regions showing LTP and LTD are likely to represent these channels by processing MFs incoming activity in different ways. Relying on the available data on synaptic plasticity modification of MF–granule cell synaptic properties, the LTP channel would be characterized by reduced response latency and increased post-synaptic firing frequency; the opposite is expected in the LTD channel (Nieus et al., 2006). Interestingly, considering electrophysiological and simulation modeling data (Mapelli et al., 2010; Solinas et al., 2010), the LTP channel is expected to display a heightened high-frequency transmission gain than the LTD channel. This prediction has not yet been experimentally confirmed.
3. A third and distinct effect of Golgi cell feed-back inhibition is the transformation of asynchronous granule cell activity into synchronous low-frequency GL oscillations. When asynchronous granule cell activity is received on their dendrites, it is summed up until the Golgi cells make a spike, which inhibits a large GL area. This results in self-sustained oscillations. And since Golgi cells, thanks to reciprocal connection through gap-junctions and inhibitory synapses, tend to form a functional syncytium, their pulsation tends to synchronize. Finally, the circuit time constant and the intrinsic resonant frequency of granule and Golgi cells will phase-lock the oscillation toward the theta band. These oscillations have been observed *in vivo* (Pellerin and Lamarre, 1997; Hartmann and Bower, 1998) and their mechanism has been predicted by computational models (Figure 8; Maex and De Schutter, 1998; Solinas et al., 2010; Casali et al., 2019).

**Figure 7:**
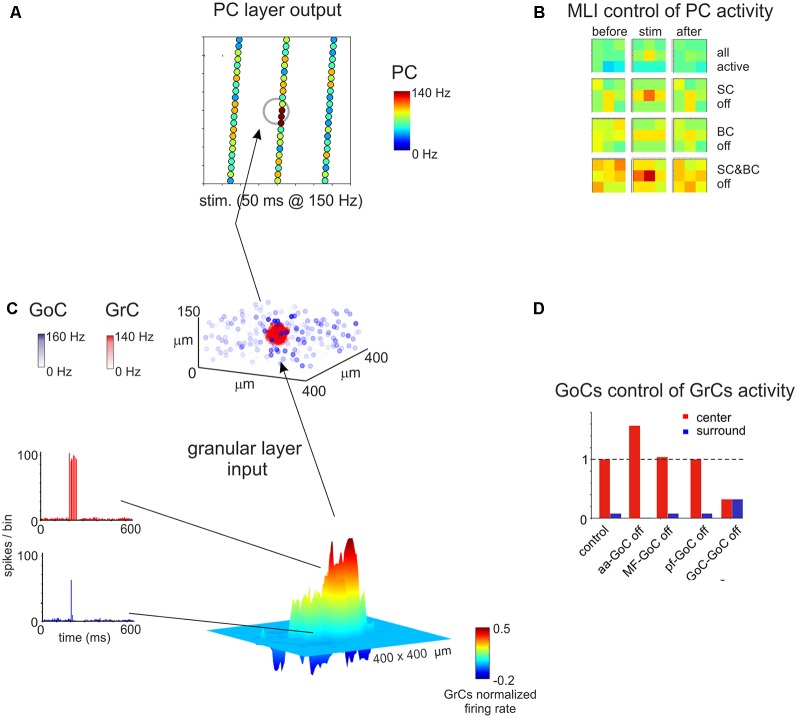
Spatial organization of the granular and ML activity predicted by computational modeling. **(A)** The map shows the activity change of PCs in response to a MF burst. **(B)** The PC activity was averaged into 3 × 3 matrices in order to better appreciate when alternative patterns are generated. **(C)** In response to a MF burst, the GL responds with a core (red area) of activity surrounded by inhibition (blue area). The upper plot represents the activity of GoCs (blue) and GrCs (red) before and after the stimulus burst. **(D)** Peri-stimulus time histograms (PSTH) of GrCs in the center-surround. The activity in the core is characterized by robust spike bursts, while just sporadic spikes are generated in the surround. No activity changes are observed outside the center-surround structure. Adapted from Casali et al. (2019).

**Figure 8:**
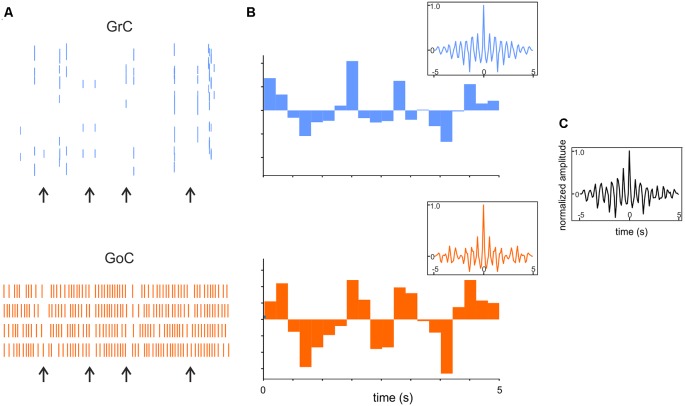
Synchronous low-frequency oscillations in the GL revealed by computational modeling. **(A)** Raster plots of representative GrC and GoC during 5 Hz random MF input. Note that synchronous patterns are visible (arrows). **(B)** Cumulative PSTH of the whole GrCs and GoCs population. Insets show the autocorrelograms of the activity in corresponding population. **(C)** Crosscorrelogram of GrC and GoC populations activity. Adapted from Casali et al. (2019).

#### Molecular Layer Interneuron Lateral Inhibition

Over 50 years ago, Szentágothai (1965) proposed that ML interneurons could laterally inhibit PCs by virtue the anatomical arrangement of excitation and inhibition onto PCs: the PFs (axons of granule cells) run coronally, whereas the axons of ML interneurons run sagittally (Figure 6b). Szentagothai’s suggestion give rise to the beam hypothesis: activation of a beam of lead to excitation a long row of PCs in the coronal plane and inhibition in laterally located PCs (Andersen et al., 1964; Szentágothai, 1965; Eccles, 1967, 1973; Palay and Chan-Palay, 1974). Several experiments supported this idea (Cohen and Yarom, 2000; Sullivan et al., 2005). Consistent with lateral inhibition, recent advances have reported that the activation of granule cells immediately underlying a PC evoked pure excitation in the sagittal orientation, while the activation of granule cells positioned more laterally—as far as 480 μm away—provide pure inhibition (Dizon and Khodakhah, 2011; Valera et al., 2016; Figure 6c). These findings are also in agreement with *in vivo* studies reporting that sensory stimulation excited a patch of PCs and simultaneously inhibited neighboring PCs (Gao et al., 2006). Given that voluntary movement requires the coordinated activity of muscles that have opposite functions (agonist and antagonist), one function of this lateral inhibition might be to efficiently generate reciprocal signals from the same MF synaptic input (Dizon and Khodakhah, 2011). In this perspective, the role of FFI in enhancing the temporal precision of PCs must be considered. Whether these roles are fundamental for cerebellar functions remains to be demonstrated.

## Plasticity in The Inhibitory Interneuron Network

Different forms of plastic changes in connection properties and/or in intrinsic excitability have been observed in inhibitory interneurons. Theoretical modeling of the cerebellar circuit suggested that plasticity in Golgi cells and ML interneurons would critically impact cerebellar circuit processing (e.g., affecting temporal precision, strength of excitatory transmission and filtering). To date, the main forms of plasticity involving cerebellar inhibitory interneurons are the following. A form of LTD has been observed at the connection between PFs and Golgi cells, following high-frequency activation (Robberechts et al., 2010). Golgi cells have also been described to undergo an increase in intrinsic excitability, as an increase in spontaneous firing, following hyper-polarization (Hull et al., 2013). In the ML, a recent study *in vivo* showed a long-lasting decrease in spontaneous firing in MLIs after theta-sensory stimulation (a pattern that is able to induce plasticity *in vivo* in the cerebellar network; Ramakrishnan et al., 2016). Other forms of LTP and LTD had been previously described *in vitro*, namely a postsynaptic PFs–ML interneurons LTD following high frequency activation of the terminal (Soler-Llavina and Sabatini, 2006), a PFs–ML interneurons LTP requiring SCs depolarization during terminal activation (Rancillac and Crépel, 2004); and *in vivo*, as a PFs–ML interneurons LTP depending on CFs simultaneous activation (Jörntell and Ekerot, 2002). For a comprehensive review of the plasticity sites in the cerebellar network and the effects of distributed plasticity on circuit processing, see Mapelli et al. (2015). Further forms of plasticity at synapses impinging Golgi cells have been predicted by theory (Garrido et al., 2013) but remain to be demonstrated experimentally.

## Insight from Detailed Cerebellar Microcircuit Models

Modeling of cerebellar function has its roots back to the work of Eccles, Marr and Albus in the second half of the 20th century (Eccles et al., 1967; Marr, 1969; Albus, 1971). The initial models were not realistic, also because available information about neuronal mechanisms was very limited. More recently, the availability of a huge amount of literature on cerebellar neuron properties and the development of high-performance computing and modeling platforms has allowed generating models incorporating fine details on intrinsic electroresponsive properties of neurons and synapses (D’Angelo et al., 2016). In the last decades, different models of cerebellar neurons have been proposed, mainly for principal cells [as granule cells (Solinas et al., 2010); and PCs (Masoli and D’Angelo, 2017)], but also for interneurons. Modeling of interneurons is indeed necessary to understand microcircuit dynamics and reproduce complex network behaviors in large-scale simulations (Figure 7; Casali et al., 2019).

### Golgi Cell Models

The first realistic model of the Golgi cell was proposed in 2007 (Solinas et al., 2007a,b) and its properties were incorporated in a detailed model of the GL later on Solinas et al. (2010). These models were able to reproduce complex non linear Golgi cell properties, as pace-making activity, phase-reset and resonance, and to help explain how these features play a role in emerging microcircuit properties (e.g., the center-surround and synchronous low-frequency oscillations in the GL; Maex and De Schutter, 1998; Solinas et al., 2010; Casali et al., 2019). The implementation of dendritic gap junctions in the realistic model showed that depolarization of one Golgi cell had an impact on the firing of neighboring Golgi cells (Dugué et al., 2009; Vervaeke et al., 2010) and enabled synchronization between Golgi cells (van Welie et al., 2016), prompting further investigation on Golgi cells mutual inhibition. For a comprehensive review of this subject see (D’Angelo et al., 2013). Recently, a simplified model of Golgi cell was obtained, maintaining the crucial firing dynamics shown in the previous models, making it feasible to integrate these properties in large-scale simulations (Geminiani et al., 2018). The 3D connectivity of Golgi cells within the granular and ML has allowed to explain also the center-surround organization of responses emerging following MF bundle stimulation (Solinas et al., 2010; Casali et al., 2019).

### Molecular Layer Interneurons Models

Realistic models of stellate and BCs, based on their experimentally-measured passive properties, are not available yet. Simplified models have been used to explain the role of these interneurons in determining PCs firing within the feed-forward loops, mimicking the irregular firing observed *in vitro* for both PCs and ML interneurons (Santamaria et al., 2007; Lennon et al., 2014). These models also suggested the importance of ML interneurons mutual inhibition to reproduce experimental data. Recently, a more detailed model of ML interneurons (but lacking a distinction between stellate and BCs (Maex and Gutkin, 2017) has been proposed in order to investigate the role of these interneurons in the timing of cerebellar processing. Interestingly, they found that synaptic and electric coupling of these neurons provides the reciprocal inhibition that allows the time constant necessary for temporal integration. Electrical coupling has been suggested to reduce ML interneurons response heterogeneity, improving the signal-to-noise ratio (Maex and Gutkin, 2017). While modeling the molecular interneuron network, the para-sagittal or medio-lateral orientation of the cerebellar cortex strip reconstructed should be taken into account. Morphological details suggested that SCs and BCs show different axonal orientations, so that SCs are expected to have an impact mostly in the transversal axis (then evident in medio-lateral strip, also referred to as “on beam”) and BCs should affect PCs processing in the sagittal axis (also referred to as “off beam”). The effect of disconnecting selectively SCs or BCs from the circuit has been described in a recent work, in a reconstruction of the scaffold model of the whole cerebellar cortex, using simplified models for single-cell neurons (Figure 7). Predictably, switching off SCs determined a spread of excitation along the PCL, prevalently along the transverse axis, while switching off BCs excitation extended mainly along the para-sagittal axis (Casali et al., 2019).

## Conclusions

The last decades have been characterized by considerable progress in understanding the diversity of cerebellar inhibitory interneurons, in terms of embryological and developmental origin, localization and distribution of neurochemical markers, morphological and intrinsic properties. The concept has emerged that neuron properties and network dynamics in the cerebellar inhibitory circuit are more complex than originally thought. Both in the granular and in the ML, cerebellar interneurons are involved in complex inhibitory chains generating feedback, feedforward and lateral inhibition that regulate spatio-temporal dynamics of fundamental importance to determine the processing capabilities of the cerebellar cortex. Interneuron inhibition leads to the emergence of: *(1) center-surround organization in the GL; (2) gain and timing regulation in the GL; (3) synchronous low-frequency oscillations in the GL; (4) beam organization in the ML; (5) burst-pause regulation in PCs; and (6) gating of synaptic plasticity*. Since the spatiotemporal pattern of cerebellar cortical activity is *de facto* controlled by cerebellar cortical interneurons, these cannot anymore be considered “subordinates” to excitatory cells (i.e., just maintaining the E/I balance) but rather integral parts of diverse microcircuits for multimodal information processing (Casali et al., 2019). This emerging view prompts for further investigations on these inhibitory interneurons in cerebellar physiology and pathology. It has already been shown that perturbing inhibitory interneurons functions results in altered cerebellar computation and motor behavior both in the GL (Watanabe et al., 1998) and in the ML (Rowan et al., 2018). Moreover, synaptic inhibition has been proposed to play a key role in neurodevelopmental disorders, such as autism, where the correct balance between excitation and inhibition might be disheveled by a malfunctioning of inhibition [as already reported for other brain regions (Pizzarelli and Cherubini, 2011)]. Future investigations should aim at dissecting the role of cerebellar cortical interneurons in specific processing features, thus shedding new light on the understanding of cerebellar processing and the generation of a unified theory of cerebellar functioning. In this perspective, the development of large-scale theoretical models will be fundamental to integrate the different neuronal types in a scaffold of the cerebellar cortex (D’Angelo et al., 2016; Casali et al., 2019). This will not only help identifying the contribution of inhibitory interneurons to local and global network dynamics but also to make predictions about their contribution to cerebellar processing and about the effects of their alterations in cerebellar pathology.

## Author Contributions

FP organized and wrote the manuscript. LM prepared the figures and contributed to writing the text. ED’A contributed to the final version of the manuscript.

## Conflict of Interest

The authors declare that the research was conducted in the absence of any commercial or financial relationships that could be construed as a potential conflict of interest.

The reviewer CH is currently the co-Field Chief Editor of Frontiers in Cellular Neuroscience with one of the authors, ED’A, and confirms absence of any collaboration to the handling editor.
